# Chemokines: humble yet mighty players in the tumour microenvironment

**DOI:** 10.3389/fimmu.2025.1601756

**Published:** 2025-08-07

**Authors:** Hima Xavier, Athira Gireesh Moly Gireesh, Juvin Ann Thomas, Priya Suboj, Arya Suresh, Emmanuel Biju, Arya Baby, Roshin Thomas Dominic, Suboj Babykutty

**Affiliations:** ^1^ Centre for Tumour Immunology and Microenvironment, Department of Zoology, Mar Ivanios College, Thiruvananthapuram, Kerala, India; ^2^ Department of Biotechnology, CEPCI Laboratory and Research Institute, Kollam, India; ^3^ Department of Botany and Biotechnology, St. Xaviers College, Thiruvananthapuram, Kerala, India

**Keywords:** tumour microenvironment, chemokines, immune surveillance, immunotherapy, angiogenesis

## Abstract

Chemokines are tiny chemotactic cytokines which play a crucial role in pathophysiology by maintaining homeostasis and inflammation. Their role in the tumour microenvironment is very much puzzling because of both pro- and anti-tumourigenic effects. Chemokines have gained much attention today, since it has been recognized that they are game changers in the TME via controlling immune cell recruitment, angiogenesis, metastasis, tumour growth and drug resistance. In this review, we are exploring the role of several chemokines and their receptors in the TME with special focus on immune cell recruitment, immune surveillance, regulation of immune checkpoints and epithelial mesenchymal transition. We are also reviewing the possibility of targeting chemokines along with immunotherapy for better outcome and disease-free survival. A better understanding on the dual role of chemokine in the TME might help to implement novel therapeutic interventions and adopt precision in targeted therapy.

## 1 Introduction

Cancer, a comprehensive disease with a progressive lurch of immunologic, metabolic, neuroendocrinal and microbial features, remains a significant economic and social burden for both developed and developing countries (1). The disease is not merely a cluster of cancer cells; rather, a collection of infiltrating as well as host immune cells along with stromal cells, fibroblast cells, extracellular matrix and blood-lymphatic vascular networks, collectively called the tumour micro environment (TME) (2). In fact, hypoxic, acidic, and immune/inflammatory TMEs are enriched with non-cellular components such as cytokines, chemokines, growth factors and metabolites (3). The interaction of tumour mass with this surrounding milieu will mediate the tumour development, immune surveillance and life-threatening distant metastasis (4). According to global cancer statistics by international agency for research on cancer - GLOBOCAN 2022, lung, colorectal, liver, breast and stomach cancers are the leading causes of cancer death worldwide. Female breast cancer (BC) has the highest global cancer incidence with approximately 2.3 million new cases followed closely by lung cancer, which ranks second with 2.2 million new cancer cases universally (5).

Surgery, chemotherapy and radiation therapies remain to be the foundational modalities of conventional cancer treatment strategies. Even though radiation therapy is a non-surgical way to control cancer cells, the treatment option has its own limitations including DNA fragmentation in adjacent healthier cells and serious side effects depending on the dosage (6). Conversely, surgery cannot be suggested as a complete treatment for the disease, rather impactful only when combined with other therapies (7). Among these therapies, chemotherapy is considered to be a successful choice for metastatic tumours. Adverse effects caused by chemotherapy such as myelosuppression, nausea, vomiting, diarrhoea and alopecia, affect the quality of life very much (8). Moreover, treatment-induced inflammatory microenvironment, drug resistance and activated cancer stemness will hinder the anti-tumoural activity of chemotherapy. Tumour cells with all their distinct pathologic features, subvert the normal physiological conditions including non-cellular components such as chemokines. Emphasising these components offers a more rational and potentially effective approach for cancer treatments (9). In fact, Hanahan and Weinberg state that the chemokines and their receptors have been involved in the majority hallmark processes of cancers (10). Upon binding, chemokine – chemokine receptor interaction activates intracellular signalling pathways leading to cell-cell interaction, migration (chemotaxis), coordination and regulation of the immune system by several modulations in gene transcription. Diverse and dynamically regulated expression of chemokine ligands and their receptors by TME facilitates immune cell recruitment and activation. Here the review focuses on the complex and diverse functions of chemokines within the tumour microenvironment (TME), highlighting their roles in tumour progression, immune evasion and disease prognosis as well as their anti-tumoural effects. The review also attempts to give an account on chemokines as therapeutic targets aiding in immune surveillance and tumour suppression.

## 2 Chemokines and their receptors

**Figure 1:**
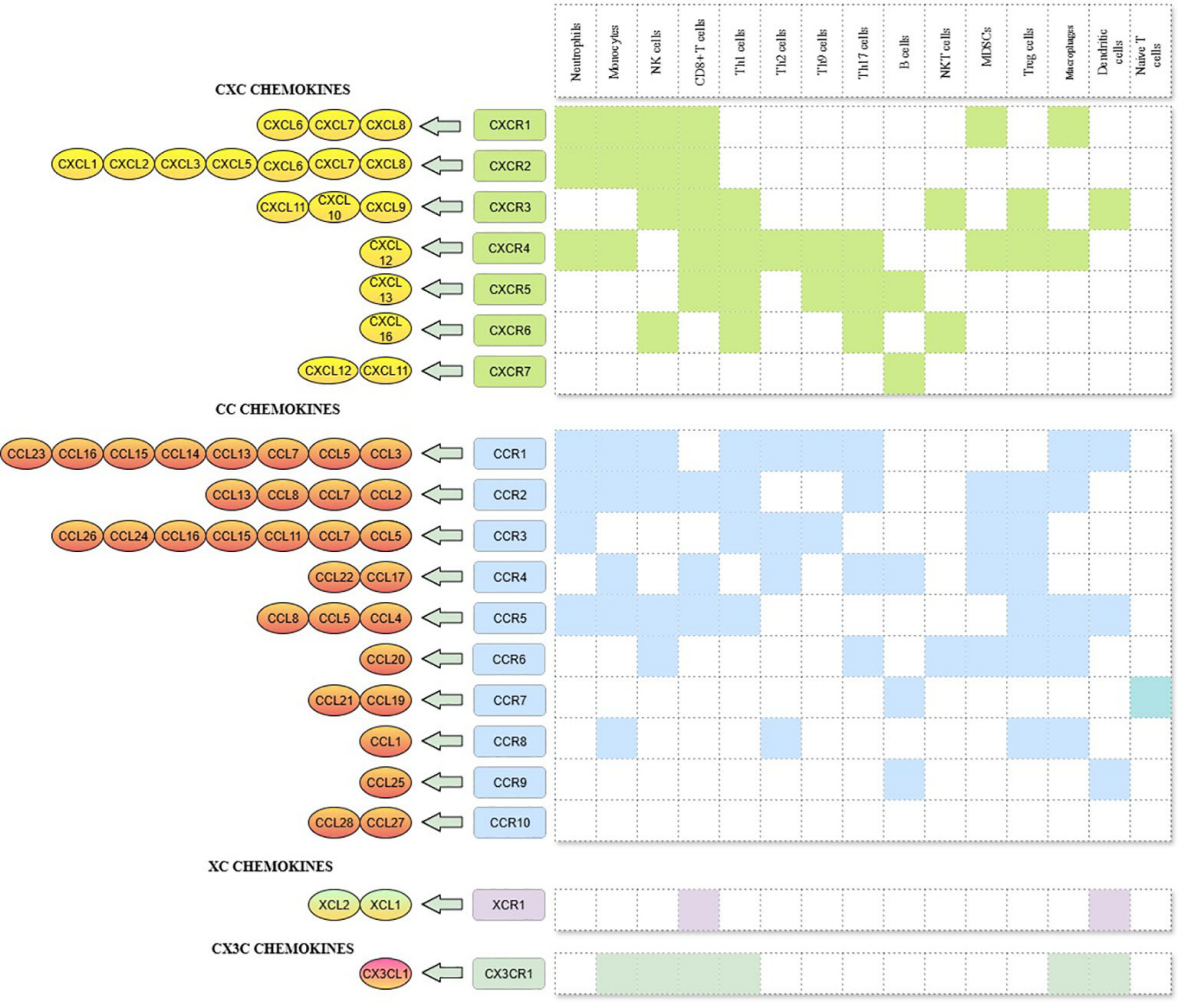
CXC, CC, XC, and CX3C chemokines, its corresponding receptors and immune cells involved in the chemokine-receptor signalling.

Chemokines are a large family of small (8–12 kDa), soluble and secretory chemotactic cytokines that regulates the migratory patterns and positioning of immune cells, specifically at the time of development, homeostasis and pathological conditions (11). The family consists of 50 chemokine ligands and 20 GPCR signalling receptors, classified into four groups - CC, CXC, CX3C and C - based on the number and location of highly conserved cysteine residues at the N terminus of the ligand, with their corresponding nomenclature designated as CCL, CXCL, CX3CL and CL for the ligands and CCR, CXCR, CX3CR and CR for the receptors (12). Chemokines and their receptors are highly compatible with each other and at the same time, both are ambiguous and are susceptible to degeneration as well I.e. Many chemokines have the binding capacity to multiple receptors and some receptors binds to many ligands. (**Figure 1**) (13). CXC chemokines are again subdivided into the ELR^+^ (CXCL1 - CXCL3, CXCL5, CXCL6, CXCL7 and CXCL8) and ELR^-^ (CXCL9 - 11) chemokine ligands based on the presence or absence of ELR (Glutamic acid - Leucine - Arginine) motif regions (14). These two subgroups execute entirely different functional roles, as follows, ELR^+^ chemokines promote angiogenesis, whereas ELR^-^ chemokines mediate anti-angiogenesis except CXCL12, which does not contain an ELR motif but exhibits angiogenic property (15).

Based on the functional role, chemokines can also be categorised into two – those which are generated and secreted constitutively to maintain homeostasis and those which are produced during pro-inflammatory stimulus to raise an immune response (16). Chemokines involved in homeostasis (e.g., CXCL12) constitutively express and mediate the migration of immune cells to the lymphoid organs, blood and peripheral tissues (17). On the other hand, inflammatory chemokines, also known as inducible chemokines, expressed only at the time of pathogenic conditions, enable the trafficking of inflammatory leukocytes to damaged tissues (18). Given the fact that chemokines recruit both pro- and anti-tumourigenic immune cells, inhibiting chemokines that mediate the recruitment of pro-tumoural immune cells and promoting the expression of chemokines which recruit anti-tumoural immune cells could be a better option in therapeutic strategies (19).

## 3 Role of chemokines in tumour microenvironment

The complex, ever-changing environment that surrounds and interacts with the tumour is called the tumour microenvironment. The cellular and non-cellular components of TME including circulatory system mediates cell survival, local invasion and metastatic dispersion (20). As compared to healthy tissues, the tumour microenvironment exhibits substantially different expression of chemokines and their receptors, facilitating the recruitment of pro- as well as anti- tumoural immune cells (21). A string of them, including, regulatory T cells (Tregs), tumour associated macrophages (TAMs), tumour associated neutrophils (TANs) and myeloid-derived suppressor cells (MDSCs) create an immunosuppressive microenvironment that allows cancer cells to evade immune surveillance and simultaneously thrive on their own (22). Alternatively, CD8^+^ T cells, CD4^+^ Th1 cells, NK cells and B cells help in the detection and elimination of tumour cells through immune surveillance and contribute to tumour elimination (23). In this segment, we are attempting to describe the pro-tumoural and anti-tumoural roles of chemokines in the TME.

### 3.1 Pro-tumoural effect

Chemokines play a crucial role in recruiting immune cells into the TME and shaping it into a pro-tumourigenic state. An aberrant chemokine profile in TME facilitates the infiltration of immune suppressive pro-tumourigenic cells into tumours such as Tregs, MDSCs, TANs and TAMs. Chemokine-mediated immune modulatory pathways are also often altered upon oncogenic transformation. Some of these cells and signalling proteins basically show both pro- and anti-tumourigenic responses. However, when they interact with the tumour progressive-immunosuppressive cells, they will transform into a pro-tumourigenic response (24). Besides immune evasion, chemokines also play a significant role in angiogenesis, metastasis, epithelial-mesenchymal transition, cancer stemness and drug resistance to keep the TME in a pro-tumoural state. Taking one example, CXCL8 and CXCL12 upregulate VEGF leads to the process angiogenesis. Furthermore, some chemokines can also accelerate angiogenesis by recruiting angiogenic factors producing leukocytes such as macrophages to the TME (25). This section elaborates on the key roles of chemokines in shaping a pro-tumoural microenvironment.

#### 3.1.1 Immune evasion and infiltration of immune suppressive cells

The complexity of immune escape mechanisms is the major hindrance to the effectiveness of cancer therapies. The initiation of anti-apoptotic pathways, activation of immune checkpoint pathways and dysfunction and exhaustion of immune systems enhance the process of immune evasion (26). Immune suppressive factors namely, chemokines, cytokines and inflammatory factors expressed by immune cells, promote the tumour immune invasion and metastasis. For instance, Tregs, which abundantly express CCR4, CCR8 and CCR10, were directed to hepatocellular carcinoma (HCC) sites, where their corresponding ligands, CCL22 and CCL28, were present, affecting the treatment outcome by evading the immune system (27). Similarly, the increased recruitment of MDSCs by the expression of CXCL2 inhibits the action of CD8^+^ T cells and promotes tumour growth in ovarian cancer (28). Tumour associated macrophages is another kind of non-tumour stromal cells, that support tumour growth and immune evasion by secreting factors such as TGF-β, VEGF, FGF-2, and various matrix metalloproteinases (MMPs). Interactions like CCL2-CCR2 axis contribute to the recruitment of macrophages to the tumour milieu. The role of chemokine – chemokine receptor interactions in the recruitment of key immune cells involved in the immune evasion will be reviewed in the following sections.

##### 3.1.1.1 Regulatory T cells

Tregs, a specific subset of CD4^+^ CD25^+^ FoxP3^+^ T lymphocytes, suppress the anti-tumour immune responses and stimulate tumour growth (29). The suppressive mechanisms mediated by Tregs includes, secretion of immunosuppressive cytokines such as IL10, TGFβ, and IL35 (30) and inhibition of dendritic cells’ functions like ability to costimulate naïve T cells by CTLA4 expression (31). The chemotaxis of Tregs to the TME driven by the CCL17/CCL22-CCR4, CCL1-CCR8, CCL28-CCR10, and CXCL9/10/11-CXCR3 axis has also been a widely studied concept among cancer biologists (32). The hypoxic tumour microenvironment upregulates the expression of CCL28 in many tumours and thereby promotes the infiltration of CCR10 expressing Treg cells, which secrete vascular endothelial growth factor A (VEGF-A) and promote angiogenesis and metastasis (33). To explicate the role of CCL28 in the hypoxia-induced Treg recruitment, *Ren, Li, et al.* blocked the CCL28 interaction by knockdown and anti-CCL28 antibody treatment, resulting in the reversion of hypoxia-induced Treg recruitment (34). CCL17/CCL22-CCR4 axis is also a well-documented pathway which leads to the accumulation of Tregs in tumours in squamous cell carcinoma (35). Blockade of CCL1-CCR8 axis is being explored as a therapeutic option to control the accumulation of CCR8^+^ Treg cells (36). CXC chemokines also play a vital role in the Treg infiltration. In particular, CXCR3, a key chemokine receptor, takes part in the recruitment of activated Treg cells to human colorectal cancer (CRC) tissues overexpressing their cognate ligand, CXCL11 (21, 37). Interferon γ produced by CD8 ^+^ helps to secrete CXCL9 and CXCL10 from the stromal cells and leads to the recruitment of CXCR3^+^ Treg cells to the TME (38). Additionally, in bone metastasis of prostate cancer, the elevated level of Treg cells with a memory phenotype is observed, along with the presence of functional CXCR4 receptors. The abundance of Tregs in metastasised bone marrow tumours create an immune suppressive tumour microenvironment (39). Thus, the chemokines, as mediators in the influx of regulatory T cells act as a therapeutic target with their pro-tumoural, immune suppressive characteristics.

##### 3.1.1.2 Tumour associated macrophages

Macrophages that infiltrate into tumour tissues or reside within the microenvironment of solid tumours are referred to as tumour associated macrophages (40). Their ability to show different and opposing phenotypes in response to environmental cues is embedded within known “plasticity,” which leads to the polarisation of undifferentiated macrophages (M_0_) to classically activated macrophages (M_1_) and alternatively activated macrophages (M_2_). Even though M_1_ and M_2_ populations exhibit anti-tumoural and pro-tumoural behaviour, respectively, TAMs primarily show a pro-tumourigenic M_2_ phenotype (41, 42). Considering the role of chemokines in TAM recruitment, the inactivation of retinoblastoma (RB), a tumour suppressor gene, enhances the secretion of CCL2, promoting tumour microenvironment by the recruitment of TAMs (43). Similarly, the blockade of CCR2 restored anti-tumour immunity in the preclinical pancreatic ductal adenocarcinoma by decreasing the number of tumour associated macrophages (44). CCL5 neutralisation in the BC cells significantly reduces the cell migration mediated by lactate activated TAMs, further strengthening the role of chemokines in developing a pro-tumoural microenvironment (45). CCL20 attracts more immature myeloid cells and differentiate into TAMs (46). Similarly, CXCL12, secreted by cancer associated fibroblasts, also recruits macrophages to the TME and promote more M_2_ polarisation (47).

##### 3.1.1.3 Myeloid-derived suppressor cells

Myeloid-derived suppressor cells, a population of immature myeloid cells with immune suppressive functions, imply adverse clinical consequences and poor survival rates in many tumours (48). According to markers and morphology, MDSCs are sub-grouped into neutrophil like polymorphonuclear-MDSCs (PMN-MDSCs) and monocyte like monocytic-MDSCs (M-MDSCs) (49). Chemokines are involved in the MDSCs migration to the tumour microenvironment (50). The overexpression of CCL2 chemokine in tumours like glioblastoma, bladder, prostate, breast, ovarian, gastric, melanoma and renal cell carcinoma facilitates the recruitment of CCR2^+^ LY6C^+^ M-MDSCs, causing a higher tumour load and reduced overall survival (49, 51). Similarly, CCL15 secreted from the xenografted orthotopic CRC model recruits CCR1^+^ MDSCs and leads to aggressive tumour growth (52). The transcription factor SNAIL upregulates the expression of CXCL2, eventually increasing the recruitment of MDSCs into the TME, highlighting the role of CXC chemokines in the MDSCs recruitment (53). Aligned with this, Liu et al. demonstrated that CRIP1 facilitates MDSC trafficking in pancreatic ductal adenocarcinoma by promoting NF-κB/p65 translocation, further establishing the role of molecular drivers in the developing an immunosuppressive tumour microenvironment (54). Post androgen deprivation therapy in prostate cancer shows significant upregulation of certain chemokines such as CXCL8 and CXCL15, which facilitates the recruitment of PMN-MDSC to the tumour microenvironment (55).

##### 3.1.1.4 Tumour associated neutrophils

Neutrophils, a plentiful constituent of leukocytes, traffic their way to the tumour tissue by local expression of chemo attractants main chemokines (56). The plasticity of tumour associated neutrophils helps to polarise neutrophils to anti-tumoural N_1_ and pro-tumoural N_2_ populations (57). Despite having differing nomenclature, N 2 TANs and Poly Morpho Nuclear -MDSCs (PMN-MDSCs) are both tumour-supporting, highly immunosuppressive neutrophil subsets that may represent the same or overlapping populations under various investigative frame works (58). The prominent neutrophil receptor, CXCR2, is responsible for the infiltration of neutrophils into tumour milieu, which regulates the involvement of neutrophils in tumourigenesis, angiogenesis and immune suppression (59). Likewise, targeted deletion of CXCR 2 in myeloid cells significantly increased anti-tumour immunity by altering the tumour microenvironment (60). The elevated expression of CXCL5 is also associated with extensive intra-tumoural neutrophil infiltration (61). In the case of many solid tumours, the potential regulatory role of TANs was mediated by the expression of elevated levels of CC chemokine receptors CCR1, CCR2 and CCR5, showcasing the contribution of CC chemokines to the TAN recruitment (12, 57, 61).

#### 3.1.2 Angiogenesis

Angiogenesis is the physiological process that mediates the development of new capillaries from the pre-existing ones through the proliferation and differentiation of endothelial cells (62, 63). Cancer cells induce neovascularisation by chemo-attractants like chemokines, leading to the formation of immature, leaky and tortuous blood vessels (64). The tumour progression with these aberrant blood vessels develops hypoxia within the core and leads to the process “angiogenic switch” by classical regulators such as vascular endothelial growth factor receptors (VEGF-R), platelet derived growth factor receptor (PDGFR), fibroblast growth factor-2 (FGF-2), interleukins and angiopoietin (63). In addition to the nutrient supply, angiogenesis is fundamental in the transition of tumours from a benign to a malignant state and eventually metastasis. Chemokines influence angiogenesis by stimulating VEGF, which in turn activate endothelial cells, which may lead to the production of PDGF, resulting in the secretion of more VEGF, leading to the advancement of this process (65). Among chemokines, the ELR^+^ motif expressed CXC chemokines such as CXCL1, CXCL2, CXCL3, CXCL5, CXCL6, CXCL7 and CXCL8, are correlated with angiogenic effect, whereas ELR^-^ chemokines like CXCL4, CXCL9, CXCL10 and CXCL11 exhibit angiostatic behaviour. In contrast to ELR^+^ chemokines, CXCL12 (ELR^-^) exert a strong angiogenic effect on the TME (12). Elevated expression of CXCL8 has been correlated with VEGF expression in BC cell lines, suggesting their crucial role in promoting angiogenesis (66). In detail, CXCL8 significantly supports the survival and proliferation of endothelial cells, facilitating the release of VEGF, subsequently promoting further proliferation and sprouting. Additionally, CXCL8 operates in an autocrine fashion by promoting the expression of MMP2 and MMP9, leading to the extracellular matrix remodelling for the process of neovascularisation in BC (67). Moreover, CXCL8 generated by endothelial cells interacts with its primary receptor CXCR2 and transactivates VEGFR2 via Src kinase-mediated receptor phosphorylation and contributes to endothelial cell permeability (68). CXCL1 plays a significant role in tumour progression and is responsible for angiogenesis by directly acting on endothelial cells in breast cancer (69). CXCL6 is primarily secreted by fibroblasts and endothelial cells and acts as a growth factor that modulates the proliferation of both endothelial cells and tumour cells, resulting in angiogenesis. Along with CXCL6, CXCL2 also exhibits the same trend in the intratumoral space, where it recruits granulocytes, thereby enhancing cancer cell survival and angiogenesis by releasing additional chemokines (2). CXCL5 has been reported to enhance angiogenesis in human non-small cell lung cancer (NSCLC) by a cyclooxygenase (COX-2)- dependent mechanism (67). Interestingly, it has been noted that IL-8 plays a more significant role than VEGF-A in endothelial cell migration. Targeted blockade of IL-8 resulted in reduced HUVEC migration even in the presence of VEGF-A, which shows the VEGF-independent angiogenic activity of IL-8 (70).

Other than CXC chemokines, several CC chemokines are also involved in the stimulation of pathological neo vascularisation, including CCL1, CCL2, CCL5, CCL11, CCL15 and CCL16. The promotion of angiogenesis by CC chemokines is mediated indirectly by employing macrophages at the site, which recruits additional pro-angiogenic chemokines and growth factors (71). CCL18, the most abundant and specific chemokine released from TAMs, plays a crucial role in the recruitment of immune cells towards tumour sites, which is essential for tumour angiogenesis and endothelial cell survival. The elevated levels are linked to a poor prognosis in BC, making CCL18 targeted therapy, a promising approach for patients resistant to anti-VEGF therapies (72). Studies suggest that when tumour cells express CCL21, an anti-tumourigenic effect occurs through the inhibition of angiogenesis and increased recruitment of CD8^+^ T cells and dendritic cells. Nevertheless, the immune response may be suboptimal due to the insufficient dendritic cell maturation (73).

#### 3.1.3 Epithelial-mesenchymal transition

A transient, reversible cellular reprogramming from an epithelial to a mesenchymal state helps in enhancing invasiveness and promoting metastasis, which is known as epithelial-mesenchymal transition (EMT) (74). Generally, the process in which the EMTs are involved is the normal embryonic development, tissue repair and wound healing. However, it is also correlated with tumourigenesis, metastasis, stemness and drug resistance (75). Cohesively arranged single layer of epithelial cells with apical-basal polarity switches to morphologically irregular, elongated, spindle-like cells with front-to-back polarity lacking cellular adhesion molecules in the process of EMT (76). Since the pathological reactivation of EMT is fundamental for tumour prognosis and metastasis, it must be tightly regulated with certain EMT-transcription factors (EMT-TFs) such as SNAIL, TWIST and ZEB (77).

Extensive research has demonstrated the oncogenic potential of chemokines to induce EMT and promote metastasis. For example, the CXCL12-CXCR4 axis mediates intracellular actin polymerisation and pseudopodia formation, thus playing a key role in EMT and cancer progression (78). Flavokawain A, a chalcone compound isolated from *Chloranthus henryi*, functionally inactivates PI3K/Akt/HIF-1α signalling pathways mediated by CXCL12 and thereby reducing the expression of a well-known EMT transcription factor, TWIST, in HCC (79). When human gastric carcinoma cells were simulated with CXCL12, their morphology changes from elongated to spindle-like fibroblast, along with decreased expression of E-cadherin and increased expression of vimentin, clearly demonstrating the relevance of the CXCL12-CXCR4 signalling axis in the process of EMT (80). CC chemokines such as CCL1, CCL2, CCL3, CCL21 and CCL25 are also associated with invasion and migration in many tumours. Upregulation of CCL8 in oesophageal squamous cell carcinoma activates the oncogenic pathway NF-κB to induce EMT (81). Also, stimulation of CCL21 in oral squamous carcinoma cells shows a reduction in E-cadherin and increase in vimentin, N-cadherin designating EMT transition (82).

#### 3.1.4 Metastasis

Metastasis is responsible for approximately 90% of cancer-related deaths, making it the leading cause of cancer mortality. It is a process by which the primary tumour smartly moves from its primary site to the surroundings and distant organs by intravasation, extravasation and localisation (83). Tumour cells alone are feeble for the development of metastasis; rather, it is executed by multiple components of the tumour niche as well as complicated cross talks between stromal and tumour cells, which further release certain chemokines and cytokines (40). For instance, mesenchymal stem cells, lymphatic endothelial cells, cancer associated fibroblasts (CAFs), MDSCs, T cells and TAMs, along with tumour cells, aid the process of cancer metastasis (84). Induction of hypoxia activates various downstream signal cascades to promote hallmarks of tumours, including metastasis (85). CAFs recruit granulocytes to the TME by producing colony stimulating factor-1 (CSF-1) that promotes tumour growth and metastasis (86, 87). Previous investigations have demonstrated that the CXC chemokine family plays a vital role in promoting tumour growth and survival, and exhibits marked metastatic potential (67). CXC chemokines are well known for their pro-tumoural activities. Among them, CXCL12-CXCR4 is one of the most researched chemokine-receptor axes considering metastatic processes (67). Giving an example, the expression level of CXCL12 in the CAFs isolated from secondary brain tumours of BC is significantly higher than in the healthier fibroblasts and CAFs from primary breast tumours (88). CXCL8-CXCR1/2 is another CXC chemokine axis in mice, activates the gene matrix metalloproteinase and positively corelates with earlier distant metastasis in BC to the bone (89). Besides, the blockade of CXCR2, a high affinity receptor for CXCL8, reduced the chance of lung metastasis by 40% in a mammalian BC model (90). CXCR12 guides premetastatic tumour cells expressing CXCR4 to intravasate into circulation through the tumour microenvironment of metastasis (TMEM). TMEM is a CXCL12-rich, immunosuppressive environment where the CD8^+^ T cell population is exhausted, despite being present in high numbers, resulting in the homing of metastatic cells around TMEM gateways (91). CXCR4/CXCL12 signalling is involved in lung carcinoma metastasis, where CXCR4 is a chemokine that is universally upregulated in tumour cells which migrate to the bone (8, 9). Nevertheless, interleukin-8, also known as CXCL8, binds to the receptors CXCR1 and CXCR2 has been shown to mediate tumour progression, epithelial-mesenchymal transition, invasion, and angiogenesis. Neutralizing IL-8 signalling with antibodies resulted in reduced bone metastasis in mice bearing MDA-MET breast cancer cells (a bone homing derivative of MDA-MB-231) (92). Apart from the CXC chemokine family, the CC family also contributes to tumour progression and metastasis. Chemokines such as CCR7, CCR9, and CCR10, have been identified as having the potential to support the metastatic seeding of cancer cells (93). Overexpression of the CC chemokine CCL2 resulted in elevated bone metastasis in MDA-MB-231 via stimulating osteoclastogenesis. Blockade of the CCL2 chemokine using a neutralizing antibody reduced bone metastasis in the MDA-MB-231 cell line (94).

#### 3.1.5 Hypoxia

In primary tumours, structurally and functionally abnormal blood vessels fail to deliver adequate oxygen, resulting in hypoxia (95). As stated by clinical studies, the measured oxygen levels in BC, cervical cancer and head and neck cancer are very less than that of normal tissues. Immunohistochemical staining of tumour biopsies manifests the increased HIF-1α protein expression, which positively mediates the expression of VEGF, Stromal cell-derived factor-1 (SDF-1) and stem cell factor and thereby induces the invasion and metastasis (96). In addition to the previously discussed roles of chemokines in cancer cell migration, invasion, and metastasis, this section examines the correlation between chemokines and hypoxia within the tumour microenvironment (TME). The chemokine axis CXCL8-CXCR1 involved in early inflammatory responses plays an important role in the migratory activity of different human breast cancer cells under hypoxic conditions. For example, IL1-β treatment under hypoxia results in the upregulation of CXCR1 by the transcriptional regulation of HIF-1α and enhances the migratory effects on BC (97). Hypoxia triggers CXCL12 expression in many tumours by the HIF-1 dependent expression of CXCL12 synthesis (39, 98). Another chemokine, CCL28 (mucosae associated epithelial chemokine) is also upregulated by hypoxia and HIF-1α in human ovarian cancer cell lines (99). Hypoxia induces the activation of CCL3 in multiple melanoma patient’s serum. These patients are also associated with worse prognosis in large B cell lymphoma (52).

#### 3.1.6 Cancer stemness

Cancer stem cells (CSCs) are considered to be one of the primary contributors of tumourigenesis, metastasis, drug resistance to conventional therapy and tumour recurrence. CSCs are undifferentiated populations such as tumour initiating cells (TICs) or tumour propagating cells (TPCs), characterised by self- renewing, multi potent and tumour initiating properties. They are tightly maintained by TME (100, 101). *Al-Hajj et al.* had first reported about the importance of tumour initiating CSCs in BC. This is also known as the first identification of CSC population in solid tumours (102). Identification of cell surface markers helped the scientists in controlling the wild behaviour of CSCs when chemotherapeutic strategies failed in eradicating them (103). In addition to surface marker targeted therapies, manipulation of TME to destroy CSCs is regarded as a powerful treatment method. There are a number of cytokines and chemokines which play a prime role in the modulation of CSCs. Hence, upon targeting CXCL12-CXCR4 axis by a CXCR4 antagonist – Plerixafor, disrupt homing of leukaemia stem cell (LSC) to the bone marrow niche (104). The researchers found that evasion of primary tumours and establishment of distant metastasis in Pancreatic ductal adenocarcinoma were caused by pancreatic CSCs, co-expressed with CXCR4 chemokine receptors. These CXCR4^+^ CSCs chemoattracted to the highly metastatic sites express high levels of their ligand, CXCL12, which stimulate the small GTPase called, RhoA, vital for the extra cellular matrix remodelling and leading to metastasis (105). Additionally, in the glioma stem cells (GSCs), the stem cell properties are maintained by the induction of CXCL1 and CCL2 chemokines (106).

#### 3.1.7 Drug resistance

Pharmacotherapy is recognised as one of the predominant clinical treatment strategies; however, the development of drug resistance which is an intricate process significantly undermines its effectiveness and contributes to disease progression (107). Tamoxifen, fulvestrants and aromatase inhibitors are some of the approved hormonal therapeutic agents used for the treatment of ER^+^ BCs; correspondingly, trastuzumab, lapatinib and pertuzumab are potential HER2 targeted agents (108). Several chemokines were significantly upregulated in drug resistant cells or patient samples than drug sensitive group when done *in vitro, in vivo* and clinical studies. The major chemokines involving in generating chemoresistance are CXCL8, CCL2, CCL5 and CCL20. They execute chemoresistance by the recruitment of immune cells, activation of the survival/proliferation pathways and promotion of invasion (109). In particular, TAM mediated CCL2 secretion promotes drug resistance and reduces apoptosis in BC cells through the activation of PI3K/Akt/mTOR pathway (110). By keeping a close watch on macrophages grown in the conditioned medium retrieved from tamoxifen-sensitive and resistant BC cells, *Li, Dongbo, et al.* observed that M_2_ polarisation due to TAM mediated CCL2 secretion was more apparent (110). Furthermore, CCL2-CCR2 axis recruit monocytes to TME and stimulate the release of pro-inflammatory cytokines such as IL1, IL6 and Tumour Necrosis Factor-α (TNF-α). Taken together, the transition of monocytes to TAM and secretion of pro-inflammatory cytokines activate PI3K/Akt/mTOR signalling pathways and mediate endocrine resistance in BC (41). CCL2 also exerts resistance to anti PD-L1 immunotherapy by the activation of PI3K/AKT and NF-κB signalling (111). In contrast, AKT signalling also promotes the differentiation of naïve CD4 T cells into T helper 1 (Th1), Th2, and Th17 cells and inhibits the transcription of Foxp3, a crucial transcription factor required for the development of T regulatory cells (Tregs). These scenarios highlight the dual role of AKT to modulate T cells in different ways in cancer immunotherapy (112). Another chemokine signalling axis, CCL15 -CCR1 also activates NF-κB pathway by recruiting monocytes, eosinophils and neutrophils to the tumour site ultimately inducing drug resistance (52).

**Figure 2:**
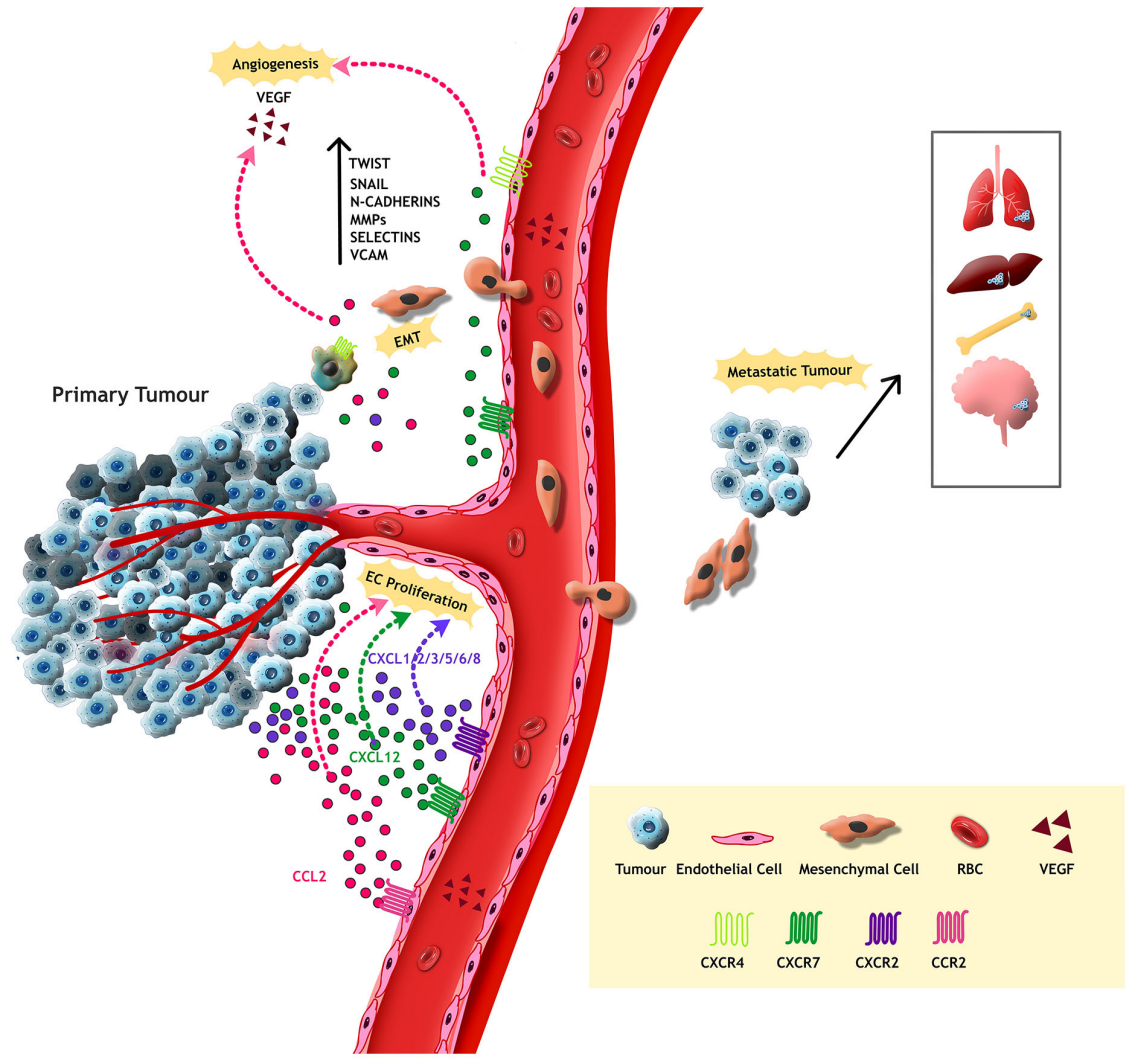
Tumour cells from the primary tumour attains mesenchymal properties through Epithelial Mesenchymal Transition, resulting in metastasis to adjacent or distant organs. Transcription factors such as Twist, Snail, MMPs and cell adhesion proteins like N-Cadherins, Selectins and VCAM assist this process. Moreover, the tumour supporting chemokines activate VEGF, thereby mediate endothelial cell proliferation and abnormal metastasis in the TME.

In conclusion, chemokines play a pivotal role in shaping the tumour microenvironment by critically regulating a wide range of processes such as immune evasion, modulation of immune cell infiltration, induction of epithelial-mesenchymal transition (EMT) and hypoxia. These functions significantly contribute to tumour progression, metastasis, angiogenesis, stemcellness and the development of drug resistance. The dual role of chemokines makes them more complex and is considered a potential strategic therapeutic target in cancer biology. Moreover, understanding the nuanced behaviour of pro-tumoural chemokines could encourage the possibility of effective therapies aimed at overcoming resistance to current treatment modalities (**Figure 2**).

### 3.2 Anti-tumoural effects

The infiltration of anti-tumoural immune cells into the TME mediate the suppression of tumour growth and proliferation. CD4^+^ T cells, CD8^+^ T cells, B cells and NK cells are the fundamental cells that activate anti-tumoural immunity (113). Apart from immune cell infiltration, the modulation of cancerous niche by polarisation of neutrophils and macrophages to their anti-tumoural phenotype also facilitate the reduction of tumour growth and proliferation (114). When chemokines bind to their cognate receptors on targeted cells, they trigger cellular signalling pathways to recruit and organise pro- as well as anti-tumour immune cells (115). The ability of certain chemokines in mediating anti-tumoural activity would be appreciable in developing novel, targeted therapeutic strategies (20). The anti-neoplastic nature of chemokines will be emphasised in the following segment.

#### 3.2.1 Chemokines in the modulation of anti-tumour microenvironment

Understanding the mechanism involved in the localisation and effects of anti-tumourigenic chemokines within the TME would be a great contribution to the field of immunotherapy (19). Cancer cells functionally sculpt their microenvironment by recruiting immune cells through the expression of various cytokines and chemokines (103). Tumours with the elevated CD8^+^ T cell infiltration and high tumour clearance capacity coupled with escalated pro-inflammatory cytokines can suppress the growth of tumours are referred to as immunologically “hot tumours” (116). Interestingly, chemokines such as CXCL9, CXCL10 and CXCL13 are involved in fostering the hot tumour milieu (117). Notably, CXCL9-CXCR3 axis stimulates the induction of apoptosis via the recruitment of CD8^+^ T cells, which produce granzyme B and perforin (118). Here, an attempt has been made to describe how the infiltration and polarisation of various immune cells modify TME.

##### 3.2.1.1 CD8^+^ cytotoxic T lymphocytes

Activated CD8^+^ T cells are the mainstay of anti-tumour immunity, resulting in effective tumour immunotherapy. Cytotoxic T Lymphocytes (CTLs), a short-lived effector cell differentiated from the naïve CD8^+^ T cells, aim at killing potentially harmful intruders in the body like viruses, foreign antigens as well as tumour cells by apoptosis through the secretion of perforins and granzymes (119). In this section, we are exploring the significance of chemokines in the recruitment of CTLs in creating an anti-tumoural microenvironment. Intratumoural expression of anti-tumoural chemokines such as CXCL9, CXCL10 and CCL5 were reported in patients undergoing chemotherapy for multiple cancer types such as melanoma, non-small cell lung cancer (NSCLC) and BC (20). Higher levels of CXCL10 in NSCLC is associated with better prognosis by the recruitment of CXCR3^+^ CD8^+^ T cells to the tumour sites (120). Another study illustrated the involvement of CXCL9 in the recruitment of CD8^+^ T cells to the TME of melanoma mouse model providing effective anti-tumoural response (121). CXCR6 expressed on cytotoxic T cells helps in the positioning of CD8^+^ T cells crucial for the effective anti-tumour responses. CXCL16- CXCR6 deficiency impairs the efficacy of cancer vaccines by reducing CD8+ T cell resident memory cells (122). CC chemokines such as CCL3 and CCL4 secreted by CD8^+^ T cells helps to make a positive feedback loops to enhance recruitment and activation of T cells (123). Another chemokine- chemokine receptor axis involved in the anti-tumoral activity of CD8^+^ T cells is CCL9/CCL21-CCR7. These chemokine axis signalling helps to organise and maintain CD8^+^ T cell pool in the TME (124).

##### 3.2.1.2 CD4^+^ T cells

CD4+ T cells play an indispensable role in anti-tumour immune responses, making them particularly significant in tumour immunology and immunotherapy. Several cytokines, specifically chemokines streamline the differentiation of CD4^+^ T cells to phenotypically distinct anti-tumoural subsets like Th1, Th17 and T follicular helper(Tfh)cells (125, 126). CXCR3, a common receptor for CXCL9 and CXCL10 recruits Th1 cells to the TME, suppressing tumour growth. Equally, upregulated expression of the receptor facilitates differentiation of naïve CD4^+^ T cells to anti-tumoural Th1 cells (127). Another fascinating study on the role of chemokines in recruiting antigen presenting cells revealed that CCL9/CCL21-CCR7 axis enables the entry of naïve CD4^+^ T cells into the tumour-draining lymph node (TDLNs), while CCL5-CCR5 facilitates their interactions with DCs (20). Another CC chemokines such as CCL19 and CCL21 facilitates CCR7+ CD4+ T cell recruitment and facilitates T cell priming in the tertiary lymphoid structure (128).

##### 3.2.1.3 Macrophages

Among the total leukocyte tumour infiltrate, tumour associated macrophages are the most abundant population. The M1 subpopulation of TAMs promotes pro-inflammatory and anti-tumour immune responses, supported by the expression of TNF-α, IFN-γ, and iNOS (129). M_2_ population promotes tumour growth, metastasis and poor prognosis. Growing evidence suggests that, rather than focussing on the depletion of TAMs in TME, the ideal therapeutic strategy is to promote the repolarisation of M_2_ phenotype to M_1_, which is anti-tumoural phenotype (130). In an independent study by *Ruytinx, Pieter, et al.*, it is demonstrated that the blockade of CCR5 induces the repolarisation of M_2_ phenotype to anti-tumoural M_1_ via the increased level of STAT-3 transcription factor (131). Similarly, another chemokine CCL5 also promotes M_1_ polarisation through the activation of oncogenic signalling pathways such as MAPK and NF-κB. The study was confirmed by downregulating CCR1/CCR5 receptors, reversing the action, underlining the role of CCL5 in anti-tumoural M_1_ macrophage polarisation (132).

##### 3.2.1.4 Neutrophils

Polymorphonuclear leukocytes/neutrophils are the first immune cells reaching at the sites of inflammation. As discussed earlier, neutrophils exist in anti-tumoural N_1_ and pro-tumoural N_2_ phenotypes according to the presence of surface markers and the role in regulation of tumour and inflammation. Here in this section, we are scrutinising intricate processes of TAN polarisation influenced by chemokines signalling pathways (133). When evaluating the response to immunotherapy in HCC patient samples, it was identified that the upregulation of CCL21 and associated TAN-N_1_ polarisation (increased expression of N_1_ neutrophil markers such as FAS, NOS2 and TNF-α) are positively correlated with better prognosis (134). Suppression of pro-tumourigenic effects by inhibiting the recruitment of neutrophils is another possible strategy to modulate TME. In addition, overexpression of CXCR2, a key chemokine receptor in many tumour is linked to poor prognosis. However, the inhibition of CXCR2 signals promote polarisation of N_1_ anti-tumoural phenotype thereby providing better prognosis and overall survival (135). Inhibition of CXCR1/2 using SX-682 decreased infiltration of MDSCs and subsequently enhanced NK Cell-based immunotherapy in head and neck tumour models (136). In line with this, Kwong et al. found that targeting the IL-8/CXCR2 axis in myeloid cells helped to overcome the resistance in hepatocellular carcinoma towards immunotherapy by reprogramming the tumour microenvironment (137). Numerous studies revealed that chemokines such as CXCL1, CXCL2, IL-8 (CXCL8), CXCL5, CXCL12, CCL2, and MIP-1α (CCL3) promote the infiltration of neutrophil to the tumoural microenvironment (61).

#### 3.2.2 Enhancement of immune check point inhibitor efficacy

Immune checkpoint pathways typically activate to prevent autoimmunity by inducing T cell anergy and exhaustion. Tumour cells hijack these checkpoint pathways to evade immune destruction by inhibiting the activity of activated T cells within the tumour microenvironment (113). Nevertheless, immune checkpoint inhibitors (ICI) reinvigorate the anti-tumour immune responses by blocking checkpoint inhibitory proteins and there by activating T cells in the TME. The advancement of therapies with ICIs are revolutionary milestones in the field of oncologic care (138). Carefully targeting the complex intercellular signalling of chemokine system combined with ICI monotherapy promises a better progression free survival and clinical improvements (139). The anti-tumour immune interventions are the result of combinatorial treatment of chemokines along with ICIs, which are achieved by escalated infiltration of T cells and inhibitions on their check points. For example, blockade of CXCL12-CXCR4 signalling along with anti-PD1 monotherapy significantly augments the anti-tumour effects. This is achieved by increasing the intratumoural CD8^+^ T cell infiltration which simultaneously inhibits the infiltration of Tregs and MDSCs (140). Recent studies on anti-PD1 monotherapy have demonstrated that CXCL9 and CXCL10 were crucial, since its receptor CXCR3 was found to be the arbitrator for the PD-1^+^ CD8^+^ T cells infiltration in B16 murine melanoma tumours (141). Similarly, another group studied on multimodal immunotherapy approach combining CXCR1/CXCR2 inhibition, neutralisation of TGF-β and blockade of PD-L1 signalling in Triple Negative Breast Cancer (TNBC). They observed a significantly higher distribution of CD8^+^ and CD4^+^ Tumour-Infiltrating Lymphocytes (TILs), which exhibit a small fraction of infiltrating CD4^+^ Foxp3^+^ Tregs indicating clinical effectiveness (142). Another ICI protein, anti-Cytotoxic T Lymphocyte associated Antigen-4 (CTLA-4) induces a stronger memory response than anti-PD-1 in tumour models (143). PD-1 and CTLA-4 inhibitory proteins are highly expressed in immune suppressive Tregs and MDSCs. The CXCL12-CXCR4 chemokine axis recruits these cells, contributing to immune suppression and resistance to immune checkpoint blockade (ICB) therapies in colon cancer. The chemokine axis activates downstream signalling pathways such as PI3K/AKT and MAPK/ERK, directing the migration of colon cancer cells to distant organs like liver and lungs. Furthermore, the blockade of CXCL12-CXCR4 axis along with anti-PD-1 and anti-CTLA-4 ICB therapies promises anti-tumour responses in colon cancer, emphasising the importance of combination therapies (144). The exclusion of T cells from tumours and deactivation of pre-existing T cells, characteristic of “immunologically cold” tumours is a limiting factor for effective ICI therapies in many malignancies. These immunologically cold tumours, with complete or hardly detectable presence of lymphocytes act as a major hindrance to anti-PD-L1 monoclonal therapies (145). A study in intrahepatic cholangiocarcinoma (ICC), found that accumulation of Tregs in the TME sequestered the activated CD8^+^ T cells and T helper cells at the tumour margin. These poorly perfused and hypoxic microenvironments make ICC tumours more immunologically cold. Nevertheless, chemotherapeutic agents like gemcitabine and cisplatin, combined with ICB treatment elevate the recruitment and activation of intratumoural CXCR3^+^ CD8^+^ T cells (146).

**Figure 3:**
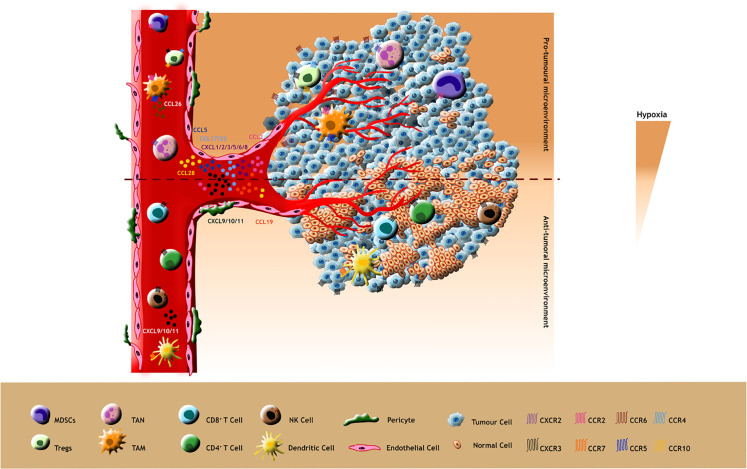
Pro-tumour effects: Pro-tumoural chemokines such as CCL5, CCL2, CCL28, CCL17/22 and CXCL1/2/3/5/6/8 released by tumour cells attract tumour promoting immune cells expressing their cognate receptors CCR5, CCR2, CCR10, CCR4 and CXCR2 respectively (Ligands and their receptors depicted as identical colours in the figure). Additionally, these immunosuppressive cells such as TAMs release pro-tumoral ligands like CCL26 and bind to their receptors in tumour cells which in turn stimulate the hypoxic TME for tumour progression. Anti-tumour effects: The less hypoxic TME promotes the anti-tumoral behaviour of chemokines making them attract immunostimulatory cells such as CD8^+^, CD4^+^, DC and NK cells. These immune supportive cells also release anti-tumoral chemokines aiding their infiltration into the TME.

Collectively, chemokine mediated modulation of TME with the surplus of infiltered anti-tumoural immune cells give rise to a better immune recognition and immune response in many tumours by various cytotoxic activities thereby enhancing immune therapeutic efficacy (**Figure 3**).

## 4 Regulation of chemokine expression in tumour microenvironment

Chemokines play a pivotal role in the elicitation of immune responses by precisely arranging leukocytes in lymphoid organs and to the sites of inflammation. These tiny yet mighty bioactive molecules influence tumour growth via multiple mechanisms either by promoting or suppressing tumour growth (147). Chemokines often recruit pro-tumourigenic immune cells such as MDSCs, TAMs, TANs and regulatory T cells etc. to stimulate the growth and proliferation of tumour cells, while recruitment of CD4^+^ T cells, CD8^+^ T cells and NK cells inhibit tumour growth, invasion and metastasis. Due to the dual hatted characteristics of chemokine molecules, their expression in the TME must be tightly regulated to prevent excessive tissue inflammation, immune cell exhaustion and damage during immune responses (148). These regulation processes include modulation of chemokine mRNA stability, sequestration of chemokines by Atypical Chemokine Receptors (ACKRs) and post-translational modifications (149). The timely downregulation of chemokine mRNA is governed by a specific mRNA instability element, adenine - and uridine rich elements (AREs) located in the 3’ UTR. Upon recognising these AREs by several RNA - binding proteins (RBPs), mRNA will undergo degradation through various mechanisms like de-adenylation, decapping and exonucleolytic decay. For example, RPL22 - an RNA binding protein, execute the degradation of CCL2 chemokine, making its expression in a controlled manner (150). Similarly, the degradation of CXCL1 mRNA is overruled by the pro-inflammatory cytokine, IL-17 signalling (151).

Atypical chemokine receptors (ACKRs), a unique subclass, functions to fine tune inflammation, immune cell migration and tissue homeostasis. The sub family includes ACKR1, ACKR2, ACKR3 and ACKR4, which are mainly expressed on non-lymphocytes but a small amount is detected in lymphocytes (152). Classic chemokine receptors switch on the downstream signalling pathways through DRYLAIF motif present in the GPCR. In contrast, ACKRs are able to regulate bioavailability of chemokines by its altered specific motifs (DRYLEIV, DRYLIST and DRYVAVT) through β-arrestin mediated non-canonical signalling pathways (153). The inability to promote cell migration along with their ability to scavenge and transport chemokines, makes them a true check point for chemokine expression (154). For instance, in NSCLCs, the tumour cells over expressing ACKR1 internalises and immobilise the bound ligands CXCL5 and CXCL8 thereby restricting the downstream signalling for angiogenesis and metastasis (155). ACKRs internalise and transport chemokines to degradative compartment, thereby controlling their concentration and bioavailability. Regulation by ACKR2, as a chemokine decoy or scavenger receptor, boosts the clearance of inflammatory CC chemokines. Inactivation of these receptors restore the secretion of inflammatory chemokines such as CCL2–5 and CCL12 in oral squamous cell carcinoma (156). In B6/129 ACKR2-deficient mice, treatment with tetradecanoyl phorbol acetate/dimethylbenz(a) anthracene (TPA, a tumour promoting agent for cutaneous malignancy/DMBA) caused elevated levels of CCL3, leading to inflammation and recruitment of polymorphonuclear cells (PMNs) and CD3^+^ T cells into papillomas. This increased inflammatory response promoted papilloma formation and made ACKR2-deficient mice more susceptible to keratinocytes proliferation. However, transgenic expression of ACKR2 in keratinocytes reduced inflammation and lowered the risk of skin tumours. These finding suggests that ACKR2 helps suppress the skin cancer by reducing chronic inflammation, likely through scavenging of CCL3 (156). The different subclasses of ACKRs, their ligands, cells expressing the receptors, motifs and roles in chemokine regulation are depicted in the **Table 1**.

**Table 1:** Role of ACKRs in chemokine regulation.

Receptor name	Alternate names	Corresponding ligands	Motif sequence similar to DRYLAIV	Expressing cell types	Mechanism of action	Reference
ACKR1	DARC (Duffy Antigen Receptor for Chemokines)	CXCL1-5, CXCL6, CXCL8, CXCL12, CCL2, CCL5, CCL7, CCL11, CCL13, CCL14, and CCL17	No homologous motif present	Erythrocytes, venular endothelial cells, and tumour cells	Mediate transcytosis, act as a chemokine reservoir	(149, 152, 154)
ACKR2	D6, CCBP2 (Chemokine binding protein 2)	CCL2-5, CCL7, CCL8, CCL11, CCL13, CCL17, and CCL22	DRYLEIV	Placenta, lymphatic endothelial cells, B cells, subsets of DCs, monocytes and macrophages	Chemokine scavengers	(149, 152, 154)
ACKR3	CXCR7, RDC1 (Renal Dysfunction Candidate 1)	CXCL11 and CXCL12	DRYLIST	B cells, endothelial cells and mesenchymal cells of central nervous systems	Chemokine scavengers, Mediate transcytosis	(149, 152, 154)
ACKR4	CCRL1 (C-C Motif Chemokine Receptor-Like 1), CCX-CKR (ChemoCentryx Chemokine Receptor)	CCL19, CCL21, and CCL25	DRYVAVT	Keratinocytes, astrocytes, lymphatic endothelial cells and thymic epithelial cells	Chemokine scavengers	(149, 152, 154)

Another mechanism in the regulation of chemokines include post-translational modifications like citrullination, nitration/nitrosylation, cleavage by the dipeptidyl peptidase- CD26 and other proteases (157). Citrullination of CXCL10 by Peptidyl Arginine Deiminase (PAD) reduces the chemotactic activity of T lymphocytes helping tumours evade immune surveillance (158). The CXCR3 receptor and its ligands were truncated by CD26 (a serine protease) in some cancers resulting in malignant progression. On the other hand, the inhibition of CD26 enhances anti-tumoural lymphocyte response and effectiveness of immunotherapy (159).

The altered metabolism of tumour cells also has a role in the regulation of chemokines. The acidic pH generated by the Warburg effect enhances the expression of CXCL8 in human ovarian cancer cells (160). Lactic acid produced during aerobic glycolysis activates NF-κB signalling, which in turn induces CXCL8 expression in breast and colon cancers (39). The hormonal regulation of chemokine is another interesting process. For instance, a study in BC showed a positive regulation of CXCL12 when treated with oestrogen, while the effects were reverted when re-treated with anti-oestrogen antibodies (161). Similarly, oestrogen-induced CXCR4 protein levels in HER2- and ER- positive breast cancer lead to the enhanced cell migration (162). In prostate cancer, androgen hormone induces the activity of transcriptional regulator, ERG, which increases the expression of CXCR4 thereby enhancing the pro-tumoural effects of CXCL12-CXCR4 signalling pathway (153). Understanding mechanisms behind the regulation of chemokine expression might be a great tool in immune surveillance and controlling tumour growth.

## 5 Chemokines and immunotherapy

US Food and Drug Administration (FDA) approved the first targeted therapeutic drug, trastuzumab, an anti-HER2 monoclonal antibody for the treatment of HER2 positive BC in 1998. This was followed by the approval of tyrosine kinase inhibitor, imatinib for Philadelphia chromosome positive chronic myelogenous leukaemia in 2001 (163). To date, a variety of molecularly targeted therapeutic agents have been clinically utilised for cancer treatment (164). TME contain many factors that are known to induce tumour growth. Targeting factors like VEGF, VEFGR, Transforming growth factor-β and various immune cells have proven their clinical efficacy in the field of cancer biology (165). In 2018, the Nobel prize in physiology and medicine was awarded for groundbreaking advancement in cancer therapy, specifically the revolutionary approach of inhibiting negative immune regulation. This recognition highlighted the significance of immunotherapy in leveraging the body’s immune system to combat cancer. James P. Allison and Tasuku Honjo were instrumental in chiselling out a transformative era in this field by emphasising its potential in immunotherapy which can act as a powerful tool in the fight against various forms of cancer (166). Analysis of current data demonstrates that chemokines and their receptors can have anti- or pro-immunogenic role depending upon the immune context on TME. Neutralisation or manipulation of chemokine gradient and its expression in tumour niche provide a promising approach for the treatment of diverse types of cancers (115). This section will delve into how the regulation of chemokine expression plays a crucial role in shaping the effectiveness of cancer immunotherapy.

We know that migration and positioning of both pro- and anti-tumour immune cells directly modulate tumour growth by chemokine and its receptors making them the most crucial biomarker for tumour immunotherapy. Blocking chemokine - receptor signal axis that supports tumour growth and also promoting chemokine- receptor interactions that limit tumour growth are the two plausible ways to utilise the chemokine system as a therapeutic agent in immunotherapy (167). The chemokines exhibiting tumour promoting functions are shown in the **Table 2**. CCL3-CCR1 signal axis mediate the formation of osteolytic lesion and leads to tumour growth. CCXR721, a selective antagonist for CCR1, blocks the formation of mature osteoclast and experience a profound decrease in the tumour burden (198). The phase III clinical trial of mogamulizumab, an anti-CCR4 monoclonal antibody in the patients with cutaneious T-cell lymphoma showed a longer progression free survival and better quality of life (199). On the contrary, the enhancement of certain chemokines helps to reduce tumour growth. For example, anthracycline treatment enhances the recruitment of functional, antigen presenting, CD11b^+^ CD11c^+^ Ly6G^-^ MHCII^+^ DC cells, stimulate the cytotoxic activity of effector T cells in fibrosarcoma model (200). The major obstacle in ICI therapies is the anergy and reluctance of effector T cell infiltration to the tumour sites. Targeting some prime chemokine - receptor axes simultaneously with ICI therapies can overcome this hurdle, contributing to tumour reduction (201). A combination of anti-PD-1 and anti-CXCR4 immunotherapy modulates tumour infiltration of CD8^+^ T cells can hence promote an anti-tumour immune response and improve survival rates (202). In brief, chemokines exhibiting elevated immune cell infiltration and inhibited pro-tumoural behaviour demonstrate better therapeutic strategies.

**Table 2:** Pro-tumorigenic effects of chemokine-receptor axis in tumour microenvironment.

Chemokine	Receptor	Cancer type	Effects	References
CXC Chemokine				
CXCL1	CXCR2	Ovarian cancer, TNBC, Uterine cervix cancer, Gastric cancer, Melanoma, Lung cancer, Colorectal cancer, Oral squamous carcinoma, Hepatocellular carcinoma, Bladder cancer and Pancreatic cancer	Promote tumour proliferation, angiogenesis, inflammation, and migration/invasion	(12, 69)
CXCL2	CXCR2	Colorectal cancer, Lung cancer, TNBC, Cervical cancer and Pancreatic cancer	Promote tumour progression, angiogenesis, and metastasis	(168, 169)
CXCL3	CXCR2	Colorectal cancer, Pancreatic carcinoma, Hepatocellular carcinoma and NSCLC	Promote tumour growth, angiogenesis, and metastasis	(170, 171)
CXCL4	CXCR3	Colorectal cancer, Lung cancer, Liposarcoma and Osteosarcoma	Promote angiogenesis in some cancers (colorectal cancer)	(172)
CXCL5	CXCR1, CXCR2	NSCLC, Papillary thyroid carcinoma, Prostate cancer, Gastric cancer, Colorectal cancer, Hepatocellular carcinoma, Bladder cancer and Head and neck squamous cell carcinoma	Promote tumorigenesis, angiogenesis, inflammation, and metastasis	(173–175)
CXCL6	CXCR1, CXCR2	Lung cancer, Melanoma and Osteosarcoma	Promote tumour growth and angiogenesis; pulmonary metastasis	(176, 177)
CXCL7	CXCR2	Clear cell renal cell carcinoma, Breast cancer and Cholangiocarcinoma	Promote tumour growth	(178, 179)
CXCL8	CXCR1, CXCR2	Acute myeloid leukemia, Lung cancer, Gastric cancer, Melanoma, TNBC, mediated lung metastasis, Colorectal cancer, Oesophageal adenocarcinoma, Pancreatic carcinoma, TNBC, Prostate cancer, Bladder cancer, Head and neck squamous cell Carcinoma and Thyroid cancer	Promote tumour growth, angiogenesis, inflammation, and metastasis	(180)
CXCL9	CXCR3	Melanoma and Hepatocellular carcinoma	Promote tumour growth	(181, 182)
CXCL10	CXCR3	Multiple myeloma	Promote bone metastasis	(183)
CXCL11	CXCR3	Colon cancer	Promote tumour growth	(184)
CXCL12	CXCR4, CXCR7	Breast cancer, Pancreatic cancer, Colorectal cancer, Bladder cancer and Thyroid cancer	Promote tumour growth, angiogenesis and metastasis and lead to poor prognosis	(88)
CXCL13	CXCR3, CXCR5	Gastric cancer, Hepatocellular carcinoma, Prostate cancer and B-cell acute lymphoblastic leukemia	Promote tumour proliferation, migration, metastasis, and recurrence	(185, 186)
CC Chemokine				
CCL2	CCR2	Ovarian, Breast and Pancreatic cancer	Promote tumour growth	(187)
CCL3	CCR1, CCR5	Oesophageal squamous cell carcinoma and Ovarian cancer	Promote tumour growth, migration and invasion	(188)
CCL4	CCR1, CCR5	Oral squamous cell carcinoma and Osteosarcoma	Promote tumour growth and angiogenesis	(189)
CCL8	CCR1, CCR2, CCR3, CCR5, CCR8	Glioblastoma and Cervical cancer	Promote tumour growth	(190, 191)
CCL18	CCR8	Gallbladder cancer	Promote tumour growth, migration and invasion	(192)
CCL5	CCR1, CCR3, CCR5	Breast cancer, Glioblastoma, Hepatocellular carcinoma and Prostate cancer	Promote tumour growth	(184)
CCL22	CCR4	Hepatocellular carcinoma, Ovarian Cancer and Oesophageal squamous cell cancer	Promote tumour growth	(193)
CCL17	CCR4	Pituitary adenoma Hepatocellular carcinoma and Gastric cancer	Promote tumour growth	(194)
CCL27	CCR10	Melanoma and Myeloma	Promote tumour growth	(195)
CCL28	CCR3, CCR10	Ovarian Cancer and Breast cancer	Promote tumour growth	(192)
XC Chemokine				
XCL1, XCL2	XCR1	Hepatocellular carcinoma, Colorectal cancer, Lung cancer, Breast cancer	Promote tumour progression	(196)
CX3C Chemokine				
CX3CL1	CX3CR1	Prostate cancer, Breast cancer, Pancreatic cancer, Glioblastoma, Ovarian and Colorectal cancer	Promote tumour growth, angiogenesis and metastasis	(197)

## 6 Future perspectives

Cancer treatments are continuously evolving progressing from traditional standard care to advanced immunotherapy shaping the future of Oncology. Over the years, a wide range of treatments including surgery, radiation therapy, chemotherapy, hormonal therapy, targeted therapy, and immunotherapy have been employed in the management of cancer. Tailoring treatments like adoptive cell therapies (ACTs) are the prime clinical methods currently in progress. Chimeric antigen receptor (CAR)-T cell therapy and engineered T cell receptor (TCR) therapy are the superior treatment methods in ACTs (203). CAR-T cell therapy uses a genetically altered CAR molecule, composed of an extra cellular antigen binding domain from an antibody and T cell receptor expressing intracellular signalling domain from patients own T cell. It has shown remarkable clinical responses (204). CAR-T cells exploit the anti-tumoural behaviour of chemokines in some cancers. For example, conditionally induced expression of chemokines such as CCL9 expressed on CAR-T cells increase the anti-tumour ability by enhancing their infiltration capacity in pancreatic ductal adenocarcinoma (205). The outcome of CAR-T cell mediated therapy against solid tumours are constrained by various reasons such as inadequate infiltration of effector cells, limited proliferation and declining effect of functional CAR-T cells etc. (206). CCL4 and CCL5 are the most robust and consistent chemokines present in the infiltration of CD8^+^ T cells. Hence, the CAR molecule engineered with CCR5, the receptor for CCL4 and CCL5, reinforce their migratory ability without affecting the cytotoxicity of CAR-T cells (206). Another ongoing therapeutic option is cancer vaccines. They efficiently eliminate malignant cells by amplifying and evoking the host immune system, making them an inevitable treatment method in the field of oncology. A phase I clinical trial of dendritic cell vaccines modified with the expression of CCL21 can potentially enhance the anti-tumour responses in NSCLC by increased immune cell infiltration (207). Several studies in cell-based immunotherapy like CAR-T cell therapies and ICI therapies (anti-PD-1, anti-PDL-1 and anti-CTLA-4) have been already transferred from preclinical to phase I and II clinical trials. Clustered Regularly Interspaced Short Palindromic Repeats (CRISPR) based technologies are also under trials in many cancer treatments. Clinical trials have shown significant results in patients with refractory cancer employing CRISPR-Cas9 in combination with T cell therapy, where CAR-T cells have been programmed using CRISPR-Cas9 (208). In short, precision medicine in the area of treatment and innovative insights mediated techniques in early diagnostics could reduce side effects and increase better prognosis.

## 7 Conclusion

Emerging therapeutic strategies are increasingly shifting focus from merely tumour cells to the broader tumour microenvironment (TME), paving the way for advanced approaches such as immunotherapy. Growing scientific evidence suggest that targeting TME derived factors including chemokines hold significant potential to enhance clinical outcomes. The immune sensitive milieu manipulates the chemokines and their receptors in favouring tumour growth. On the other hand, some of them contribute to anti-tumoural properties as well. The pro-tumoural behaviour of chemokines have been well researched. The immune suppressive cells such as Tregs, MDSCs, TAMs and TANs express chemokines which chemotactically binds to their cognate receptors on tumour, stromal cells and vice versa, advocating immune evasion and malignancy. Several chemokines - receptor axes mediate the oncogenic processes such as abnormal angiogenesis, EMT, metastasis and the other hallmarks of tumour like hypoxia, cancer stemness and drug resistance. It was noted that the gut microbiome as well as intratumoural microbiota in some cancers contribute to tumour promotion through chemokines. The anti-tumoural face of chemokines are rather seen when concentrating on CD4^+^ T cells, effector CD8^+^ T cells, B cells and NK cells along with M_1_ and N_1_ phenotypes of TAMs and TANs respectively. The ability of certain chemokines in mediating anti-tumoural activity would be a breakthrough in developing targeted and cutting-edge therapeutic strategies. Given the fact that immune checkpoint inhibitor therapy is greatly employed clinically and have successful outcomes, combination therapies targeting ICIs and chemokines would be a promising strategy in immunotherapy. Despite their role in both tumour enhancement and suppression, their levels are regulated in TME by several modifications and feedback mechanisms. The new and more feasible treatment applications in the field of oncology include tailored, precision medicines, adoptive cells therapies, CART-therapies and CRISPR based options. These are all plausible methods in this area. The paradoxical behaviour of chemokines can be strategically exploited to develop innovative and effective therapeutic approaches suppressing immune evasion and tumour growth while enhancing immune surveillance and improving prognosis.

## Glossary

TME: Tumour microenvironment
Tregs: Regulatory T cells
TAMs: Tumour associated macrophages
TANs: Tumour associated neutrophils
MDSC: Myeloid-derived suppressor cells
NK cells: Natural killer cells
VEGF-A: Vascular endothelial growth factor-A
RB: Retinoblastoma
PMN-MDSCs: Polymorphonuclear- myeloid-derived suppressor cells
M-MDSCs-: Monocyte-like monocytic- myeloid-derived suppressor cells
VEGF-R: Vascular endothelial growth factor receptors
PDGFR: Platelet derived growth factor receptor
FGF-2: Fibroblast growth factor -2
PDGF: Platelet derived growth factor
MMP2: Matrix Metalloproteinase-2
MMP-9: Matrix Metalloproteinase-9
VEGFR2: Vascular endothelial growth factor receptor-2
EMT: Epithelial mesenchymal transition
EMT-TFs: EMT-transcription factors
CAFs: Cancer associated fibroblasts
BC: Breast cancer
HIF-1 α: Hypoxia inducing factor 1 α
SDF-1: Stromal cell-derived factor-1
HCC: Hepatocellular carcinoma
NKT Cells: Natural killer T cells
CRC: Colorectal cancer
CSCs: Cancer stem cells
TICs: Tumour initiating cells
TPCs: Tumour propagating cells
LSC: Leukaemia stem cell
ER^+^: Estrogen receptor +ve
HER2: Human epidermal growth factor receptor 2 Tumor necrosis factor- αTLs
PD-L1: Programmed death ligand 1
CTLs: Cytotoxic T lymphocytes
NSCLC: non-small cell lung cancer
TFH: T Follicular helper cells
DCs: Dendritic cells
TNF- α: Tumour necrosis factor-alpha
IFN-γ: Interferon-gamma
iNOS: Inducible nitric oxide synthase
STAT 3: Signal transducer and activator of transcription 3
MAPK: Mitogen-activated protein kinase
NF-κB: Nuclear factor-kappa B
NOS2: Nitric oxide synthase-2
ICI: Immune checkpoint inhibitors
TGF-β: Transforming growth factor-beta
PD-1: Programmed cell death protein-1
TNBC: Triple negative breast cancer
CTLA-4: Cytotoxic T-lymphocyte -associated protein 4
ICB: Immune checkpoint blockade
ICC: Intrahepatic cholangiocarcinoma
ACKRs: Atypical chemokine receptors
AREs: Adenine- and uridine rich elements
RBPs: RNA- binding proteins
GPCR: G-Protein coupled receptor
PAD: Peptidyl arginine deiminase
FDA: Food and drug administration
DEGs: Differentially expressed genes
CAR-T: Chymeric antigen receptor- T-cell therapy
TCR: T- cell receptor
